# The Book World of Medicine and Science

**Published:** 1900-04-28

**Authors:** 


### The Book World of Medicine and Science.

Chemistry: Its Evolution and Achievements. By Ferdinand O. Wiechmann, Ph.D. (New York: William R. Jenkins. 1899. Pp. 154.)

We feel that the author has been hampered by lack of space, and that with a wider opportunity he might have produced a volume even better than the little book before us, which is worth reading either by the specialised chemical student or by the man in the street. It is eminently readable, and its subject forces it to be interesting; the chief fault in it is an occasional lack of value in emphasis. The review of the work of the chemists of the eighteenth and early nineteenth centuries is very well done, and indicates a real knowledge of the history and a due estimate of the advances made. The history of the chemical triumphs of the last thirty years is a difficult chapter to write, and Mr. Wiechmann would have been at better advantage had his space allowed him more technical detail. To mention Bessemer's steel process without any account of the principles which underlie such an important chemical industry is to neglect the stimulation of knowledge. The early history of chemistry is somewhat scanty and confused. Our author gives a long quotation from Hortulanus (eleventh century), but seems unaware that most of the Hermetic writings are to be found embodied in Lactantius (A.D. 300), Cyrillus, Suidas, and Stobæus (A.D. 500), and that the Ebers Papyrus, discovered in 1875, is a probable representative of the original series. Englishmen so persistently miscall Bacon "Lord Bacon," that it is interesting to find Mr. Wiechmann more correctly term him "Francis Bacon of Verulam," though he ignores Bacon's peerage. The volume is admirably printed on excellent paper.

Contagious Ophthalmia, Acute and Chronic. By Sydney Stephenson, Ophthalmic Surgeon to the Evelina and to the North-Eastern Hospital for Children, &c. (London: Baillière, Tindall, and Cox. Price 2s. 6d.)

This is the first of a series of medical monographs, edited by Dr. David Walsh, the aim of which, we are told in the preface, is to sketch in brief compass the chief featu resof given subjects of everyday interest to students and practitioners. An objection to educational monographs of this character is, we think, the amount of repetition which a series of them will necessarily involve. A student will find his labours greatly increased if he tries to study from such small monographs in place of systematic text-books, although a practitioner wishing for recent information on some special point may find them of much value. In the work before us Mr. Stephenpon must be congratulated in having kept well to his subject, while at the same time he gives sufficient information on other matters bearing on it, such as the anatomy of the conjunctiva, the clinical examination of the eye and bacteriological methods, as to render what he has to say intelligible. The main feature of the book is the importance which the author attributes to the bacteriological examination of the secretions from the conjunctiva in the various forms of ophthalmia. He apparently looks forward to a day when the old classification of ophthalmia according to clinical characters will be replaced by one based upon the micro-parasite of the associated discharge. He does not fail, however, to mention certain difficulties which present themselves in forming such a classification, viz., that some of the micro-organisms associated with ophthalmia (Klebs-Löffler bacilli, pneumococci, staphylococci, streptococci) may be found upon healthy conjunctiva, and that the clinical features of an ophthalmia apparently due to the same micro-organisms vary in character. Thus, he says, the gonococcus may now and then cause an inflammation of the conjunctiva so slight as to resemble muco-purulent ophthalmia, at other times an affection looking like diphtheria, and that Weeks bacillus may depart from its usual course and set up what appears to be a purulent ophthalmia. Such variations are attributed to the age of the patient, his powers of resistance, or to the virulence of the particular microbe. Seeing, then, that a particular disease germ does not always give rise to a particular reaction, the clinical character of a case must still remain the most important factors in prognosis and treatment, and we think the author is inclined to exaggerate when he so strongly urges the paramount importance of a bacteriological examination of the secretions to determine these points. Mr. Stephenson gives a very clear account of the differences between follicular conjunctivitis and trachoma. He would rename these affections, calling the former simple folliculitis, and the latter specific folliculitis. He also points out what has often been lost sight of, namely, the extreme frequency with which follicular enlargements are met with in the conjunctiva of presumably healthy children.

Aids to Pharmacy. By A. Campbell Stark, Demonstrator on Materia Medica and Pharmacy at St. George's Hospital. (London: Baillière, Tindall, and Cox. 1 Pp. 170. Price 2s. 6d. cloth, 2s. paper.)

This is a new and revised edition of the author's "Practical Pharmacy," which is now converted in its new form into a unit of the seemingly inexhaustible "Aid" series. Advantage has been taken in this edition of revising the work in accordance with the "British Pharmacopœia" of 1898. We need hardly say that this excellent little volume is not intended to replace or take the place of any of the well-known works on materia medica; it has more the nature of a practical reference book, and, as such, leaves nothing to be desired. At the same time sufficient information may be obtained from its pages to pass the "Practical Pharmacy" of the Conjoint Board. The syllabus of the latter examination is given at the beginning of the book, and will be found very useful. Then follow short notes on pharmaceutical processes and medicinal preparations. Besides being given in a separate table, the substances that have to be recognised bythe student have an asterisk attached throughout the text; in this way the student has ever before him a reminder of the practical nature of the materia medica examination. Organic and vegetable substances with similar pharmacological properties are grouped together. A table giving the drugs and their preparations, with strengths and doses, constitutes a very useful index with which to conclude the book. We heartily recommend this little book, not only to medical students, but to all those who are engaged in dispensing, as its well-expressed and accurate information cannot fail to be useful when reference is required on a practical point.

